# Overlapping Properties of the Short Membrane-Active Peptide BP100 With (i) Polycationic TAT and (ii) α-helical Magainin Family Peptides

**DOI:** 10.3389/fcimb.2021.609542

**Published:** 2021-04-26

**Authors:** Christian Mink, Erik Strandberg, Parvesh Wadhwani, Manuel N. Melo, Johannes Reichert, Irene Wacker, Miguel A. R. B. Castanho, Anne S. Ulrich

**Affiliations:** ^1^ Institute of Organic Chemistry, Karlsruhe Institute of Technology (KIT), Karlsruhe, Germany; ^2^ Institute of Biological Interfaces (IBG-2), KIT, Karlsruhe, Germany; ^3^ Instituto de Medicina Molecular, Faculdade de Medicina, Universidade de Lisboa, Lisbon, Portugal; ^4^ Cryo EM, Centre for Advanced Materials, Universität Heidelberg, Heidelberg, Germany

**Keywords:** short multifunctional peptide BP100, cationic amphipathic α-helix, membranolytic and cell-penetrating mechanisms, antimicrobial activity, hemolysis, vesicle fusion, vesicle leakage

## Abstract

BP100 is a short, designer-made membrane-active peptide with multiple functionalities: antimicrobial, cell-penetrating, and fusogenic. Consisting of five lysines and 6 hydrophobic residues, BP100 was shown to bind to lipid bilayers as an amphipathic α-helix, but its mechanism of action remains unclear. With these features, BP100 embodies the characteristics of two distinctly different classes of membrane-active peptides, which have been studied in detail and where the mechanism of action is better understood. On the one hand, its amphiphilic helical structure is similar to the pore forming magainin family of antimicrobial peptides, though BP100 is much too short to span the membrane. On the other hand, its length and high charge density are reminiscent of the HIV-TAT family of cell penetrating peptides, for which inverted micelles have been postulated as translocation intermediates, amongst other mechanisms. Assays were performed to test the antimicrobial and hemolytic activity, the induced leakage and fusion of lipid vesicles, and cell uptake. From these results the functional profiles of BP100, HIV-TAT, and the magainin-like peptides magainin 2, PGLa, MSI-103, and MAP were determined and compared. It is observed that the activity of BP100 resembles most closely the much longer amphipathic α-helical magainin-like peptides, with high antimicrobial activity along with considerable fusogenic and hemolytic effects. In contrast, HIV-TAT shows almost no antimicrobial, fusogenic, or hemolytic effects. We conclude that the amphipathic helix of BP100 has a similar membrane-based activity as magainin-like peptides and may have a similar mechanism of action.

## Introduction

Membrane-active peptides tend to have short sequences and simple secondary structures, from which it often appears straightforward to predict their mechanism of membrane-perturbation. Various models for peptide-lipid interactions have been proposed that are intuitively appealing, such as the barrel-stave pore (Mathew and Balaram, 1983), the toroidal wormhole (Ludtke et al., 1996; Huang, 2006), the carpet mechanism (Pouny et al., 1992), and the formation of inverted micelles (Derossi et al., 1996; Afonin et al., 2006). It has also been suggested that the physical process of membrane solubilization by amphiphilic peptides can be described in terms of different routes through a complex phase diagram (Bechinger and Salnikov, 2012). However, despite the appeal of these generalizable pictures, unexpected structural results can be encountered when faced with the actual properties of a specific sequence. Here, we studied the short yet highly helical peptide BP100, which shares some striking similarities with two distinctly different classes of membrane-active peptides: (i) well-structured amphiphilic helical peptides such as magainin 2 (Zasloff, 1987), and (ii) short, highly charged cell penetrating peptides such as HIV-TAT (Vives et al., 1997; Thorén et al., 2003).

Cationic membrane-active peptides perform numerous important biological functions in cells. It is important to realize that they can be classified either according to their 3D structure or according to their functional usage, for which overlapping profiles tend to emerge. Antimicrobial peptides (AMPs), also known as host defense peptides, form part of the innate immune system of most organisms (Boman, 1991; Brogden, 2005; Peschel and Sahl, 2006). Many of them fold into amphiphilic helices and kill bacteria by permeabilizing their membranes (Boman, 2003; Papo and Shai, 2003; Fernandez et al., 2009). Cell-penetrating peptides (CPPs), on the other hand, are used to transport hydrophilic cargo into cells, preferably without damage to the plasma membrane (Langel, 2002). Some of them also have amphiphilic helical structures, while others tend to be highly charged and unstructured like HIV-TAT. In fact, many CPPs show antimicrobial activity and *vice versa*, depending on which cell type and lipid composition they encounter (Nekhotiaeva et al., 2004; Henriques et al., 2006; Strandberg et al., 2007; Wadhwani et al., 2012a). Fusion peptides (FPs), finally, are involved in specific processes where cellular membranes need to merge (Blumenthal et al., 2003; Zheng et al., 2006; White et al., 2008). Many FPs are rich in Gly and have a rather flexible structure (Reichert et al., 2007), but they also share many similarities with AMPs and CPPs (Wadhwani et al., 2012b).

Short peptide sequences, that are easy and cost-efficient to synthesize, are of special interest for therapeutic and biotechnological purposes. Much effort has been spent to systematically optimize and miniaturize naturally occurring AMPs. One such product is the 11-mer sequence BP100 (KKLFKKILKYL-NH_2_), which was developed from hybrids of cecropin and melittin (Andreu et al., 1992; Cavallarin et al., 1998; Ferre et al., 2006; Badosa et al., 2007; Ferre et al., 2009). BP100 had originally been optimized as a membrane-active AMP against plant pathogens for applications in agriculture (Badosa et al., 2007). The peptide was also found to be highly effective against many other bacteria, with the added medical benefit of causing only comparatively little hemolysis. Atomic force microscopy showed severe damage of the cell envelope of *Escherichia coli* bacteria at its minimum inhibitory concentration (MIC) of 3 µM (Alves et al., 2010). Interestingly, BP100 was also found to be taken up into tobacco cells without causing leakage (Eggenberger et al., 2011; Eggenberger et al., 2017). It was thus used as a cell-penetrating carrier to deliver various types of cargo into tobacco cells (Eggenberger et al., 2011; Gao et al., 2016). Considering this exciting multifunctional potential of BP100 towards membranes, in the present study we extend previous studies by (i) testing the ability of the BP100 to enter human cell lines, to (ii) studying the toxic side effects, and (iii) - given the significant overlap between AMPs, CPPs and FPs - checking for its fusogenic abilities.

In view of such a wide range of biological activities, a most intriguing question concerns the mode of action by which this short peptide permeabilizes/permeates/penetrates lipid membranes. Obviously, BP100 does not act as a simple detergent, as it would otherwise be highly toxic. A recent solid-state NMR and oriented circular dichroism (OCD) study showed that BP100 forms an amphipathic α-helix in the presence of micelles and lipid bilayers (Wadhwani et al., 2014). Surprisingly, the disturbance to lipid bilayers typically caused by other helical peptides was unusually small for BP100 (Wadhwani et al., 2014; Misiewicz et al., 2015; Zamora-Carreras et al., 2016). A careful analysis of its molecular orientation and dynamics in the membrane-bound state has been performed using ^2^H-, ^15^N- and ^19^F-NMR, as well as OCD spectroscopy (Wadhwani et al., 2014; Misiewicz et al., 2015; Zamora-Carreras et al., 2016). These studies showed that the BP100 helix is aligned almost parallel to the membrane surface, and that this short compact structure is highly mobile as a rigid body.

To better understand the mechanism of action of BP100, we here compare it with two classes of membrane-active peptides which have been extensively studied. Their mechanism of action has been mostly elucidated.

On the one hand, BP100 forms a cationic, amphipathic a-helix in the membrane, and is therefore similar to many well-known AMPs and CPPs. For example, magainin 2 (MAG2) from the frog *Xenopus laevis* (Zasloff, 1987) consists of 23 residues [GIGKFLHSAKKFGKAFVGEIMNS], PGLa from the same frog (Andreu et al., 1985) has 21 residues [GMASKAGAIAGKIAKVALKAL-NH_2_], and the related designed sequence MSI-103 (Blazyk et al., 2001) also has 21 residues [(KIAGKIA)_3_-NH_2_]. The mode of action of these long amphiphilic peptides is attributed to the formation of oligomeric transmembrane pores, in which the polar residues line an aqueous channel, with or without participation of charged lipid head groups (Matsuzaki et al., 1995; Matsuzaki, 1998; Grau-Campistany et al., 2015; Grau-Campistany et al., 2016).

On the other hand, the short length (11 amino acids) and high charge density (5 charges at side chains and one N-terminal), and the efficient cell uptake of BP100 is similar to some well-known and effective CPPs like for example the unstructured, short, highly cationic peptides poly-arginine, poly-lysine, or the 13 residue peptide TAT[48-60] from HIV-1 [GRKKRRQRRRPPQ] (in this paper we will just call it TAT) (Vives et al., 1997; Thorén et al., 2003). These densely charged peptides tend to be unstructured, both in solution and in the presence of membranes (Futaki et al., 2001; Eiriksdottir et al., 2010; Wadhwani et al., 2012a), and they seem to translocate spontaneously across membranes, driven by the transmembrane potential (Terrone et al., 2003; Zhang et al., 2009). It has been suggested that this process may involve a collapse of the TAT peptides into inverted micelles (Afonin et al., 2006), a cooperative electrostatic breakthrough across the lipid bilayer (Binder and Lindblom, 2003), or proceed *via* an anion-mediated route (Sakai and Matile, 2003).

**Figure 1:**
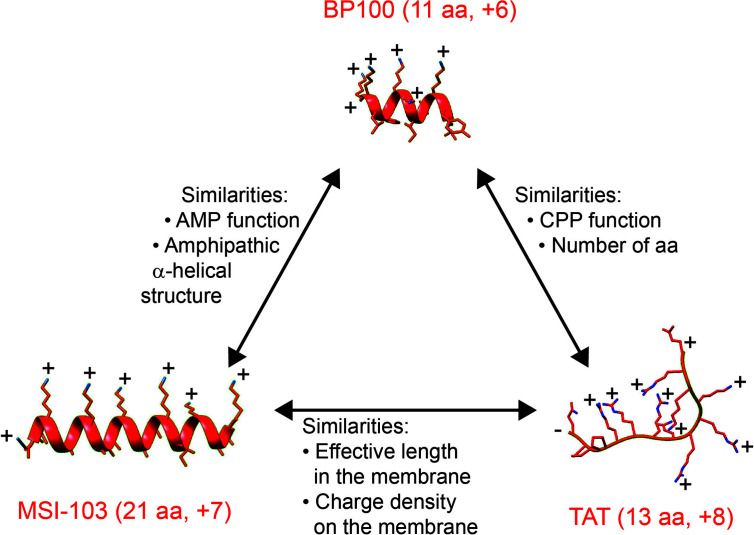
Comparison of BP100, MSI-103, and TAT, all of which have a similar net charge. On the one hand, BP100 resembles MSI-103 in having a similar AMP function and amphipathic α-helical structure, and on the other hand, BP100 has a similar CPP function and short sequence as TAT. However, these two types of representative peptides – MSI-103 and TAT – differ fundamentally in size and secondary structure. We suggest that MSI-103 and TAT may nevertheless resemble each other in terms of their similar effective length and similar charge density, when considering their respective α-helical and unstructured forms in the membrane-bound state. Peptides are shown with helices as ribbons, side chains as stick models, with N atoms in blue. Side chain and terminal charges are indicated. TAT is unstructured and highly flexible; only one of the possible and interchanging conformations is shown. [Helix figures were prepared using the 3D-HM web application (Reißer et al., 2014). The structure rendering was done using UCSF Chimera (Pettersen et al., 2004)].

**Figure 1** shows a comparison of the multifunctional BP100 with MSI-103 as a representative antimicrobial peptide on the one hand, and with the representative cell-penetrating TAT on the other hand. BP100 shares important characteristics with each of these membrane-active peptides, even though the magainin family and the TAT-type peptides are considered to represent entirely different structural classes. All three sequences carry a similar net charge. BP100 and MSI-103 both form amphipathic helices. BP100 and TAT has similar lengths. The key question in our current investigation is whether BP100 operates either like the pore forming magainin-like AMPs or rather like the densely charged TAT-like CPPs. Even though it is often assumed that a functional mechanism can be inferred from the structural properties of a membrane-bound peptide, BP100 could arguably belong to either of the two classes – or to neither of them. To get some better understanding of its biological actions and to investigate which mechanistic picture is most appropriate for BP100, we have used a range of biological assays and biophysical techniques. Here, we compare the behavior of BP100 with the well-known amphiphilic AMPs MAG2 (Zasloff, 1987), PGLa (Soravia et al., 1988), and MSI-103 (Maloy and Kari, 1995), with the designated helical amphiphilic CPP MAP (Steiner et al., 1991; Oehlke et al., 1998), with the densely charged TAT (Vives et al., 1997), and in the vesicle fusion assay also with the representative fusion peptide FP23 from HIV (White et al., 2008), as listed in **Table 1**.

**Table 1:** Sequences of used peptides.

Peptide	Sequence	Length^a^	Charge^b^
BP100	KKLFKKILKYL-NH_2_	11	+6
MAG2	GIGKFLHSAKKFGKAFVGEIMNS	23	+3/+4
PGLa	GMASKAGAIAGKIAKVALKAL-NH_2_	21	+5
MSI-103	(KIAGKIA)_3_-NH_2_	21	+7
MAP	KLALKLALKALKAALKLA-NH_2_	18	+6
TAT	GRKKRRQRRRPPQ	13	+8
FP23	AVGIGALFLGFLGAAGSTMGARS-NH_2_	23	+2

a Number of amino acid residues.

b Net charge; depending on pH, His can be protonated or deprotonated.

## Materials and Methods

### Materials

Peptides were synthesized using a standard Fmoc solid phase protocol (Fields and Noble, 1990; Wadhwani et al., 2014). For cell uptake studies, BP100 and other peptides as listed in **Table 1** were fluorescence labeled with Fluka 630 red (Sigma-Aldrich, Germany): after completion of synthesis 20 mg of dry resin was swollen in DCM, 0.5 mL of 1.5 mM activated Fluka 630 red in DMF was added and incubated overnight, then filtered, washed (three times in DMF and three times in MeOH), dried under vacuum for 24 h, and cleaved. All peptides were purified with HPLC regardless if labeled or not.

The lipids 1,2-dimyristoleoyl-sn-glycero-3-phosphatidylcholine (DMoPC), 1,2-dimyristoleoyl-sn-glycero-3-phosphatidylglycerol (DMoPG), 1-palmitoyl-2-oleoyl-sn-glycero-3-phosphatidylcholine (POPC), 1-palmitoyl-2-oleoyl-sn-glycero-3phosphatidylethanolamine (POPE), 1-palmitoyl-2-oleoyl-sn-glycero-3-phosphatidylglycerol (POPG), 1-palmitoyl-2-oleoyl-sn-glycero-3-phosphatidylserine (POPS), sphingomyelin (SM), cholesterol (CHOL), and 1,2-dioleoyl-sn-glycero-3-phosphoethanolamine-N-(lissamine rhodamine B sulfonyl) (Rhod-PE) were obtained from Avanti Polar Lipids (Alabaster, AL); 1,2-dierucoyl-sn-glycero-3-phosphatidylcholine (DErPC) and 1,2-dierucoyl-sn-glycero-3-phosphatidylglycerol (DErPG) were purchased from NOF Corp. (Grobbendonk, Belgium).

The fluorescent probes 8-amino-naphtalene-1,3,6-trisulfonic acid sodium salt (ANTS) and pxylenebis(pyridinium)bromide (DPX) were purchased from Invitrogen – Molecular Probes (Karlsruhe, Germany).

### Minimum Inhibitory Concentration (MIC) Assay

The antimicrobial activity of BP100 was tested using a standard MIC dilution assay, as previously described (Strandberg et al., 2007; Wadhwani et al., 2012a). Two Gram-negative strains, *Escherichia coli* (DSM 498) (K12) and *Enterobacter helveticus* (DSM 18396), and one Gram-positive strain, *Kocuria rhizophila* (DSM 348) were used. Bacteria were grown in Luria-Bertani medium at 37°C and 230 rpm overnight, and diluted in 1% trypticase soy broth (TSB). Peptide stock solutions (1024 μg/mL) were prepared in water. Microtiter plates (96 wells of 100 μl) were filled with 50 μl of 1% TSB, and serial 2-fold dilutions of peptides were arranged in columns. The two final rows of each plate remained without peptide, so that the penultimate data point served as the positive control (no peptide) and the final one as the negative control (not inoculated). To each well (except for the final row of each plate), an aliquot of 50 µl of a bacterial suspension containing 10^6^ colony-forming units/mL was added to obtain a final volume of 100 µL. Antibacterial activities are expressed as the MIC and correspond to the lowest concentration of peptide, in micromolar units, at which no bacterial growth was detected after overnight incubation at 37°C. The tests were made in triplicate for each peptide, and the value shown is the lowest concentration where at least for two repetitions the growth was inhibited.

### Hemolysis Assay

The hemolytic effect of the peptides was tested on human erythrocytes as previously described (Strandberg et al., 2007). Citrate phosphate dextrose-stabilized blood bags with erythrocyte suspensions were obtained from the blood bank of the local municipal hospital (Karlsruhe, Germany). Erythrocytes were washed twice with 9-fold excess of Tris buffer (172 mM, pH 7.6 at 0°C) followed by centrifugation at 600 g for 10 min at 4°C, and kept on ice in between. After the second wash, the erythrocytes were transferred from the sediment to a fresh tube with the same precooled buffer to be diluted to about 10% (v/v) hematocrit, giving the stock cell suspension, which was kept on ice. For each peptide, serial 2-fold dilutions in Tris buffer (pH 7.6 at 37°C)/dimethylsulfoxide (9:1 v/v) were prepared to have 6.25-200 µM, twice the desired end concentration (an equal volume of erythrocyte suspension will be added later to start the incubation). The stock cell suspension was further diluted to about 0.5% (v/v). After preincubating for 3 min, 200 μL of the resulting erythrocyte dilution was transferred to each tube of the corresponding peptide serial dilution, to a final concentration of 0.25% (v/v). For each dilution series, zero hemolysis was obtained by adding the erythrocytes to Tris buffer (pH 7.6 at 37°C)/dimethylsulfoxide (9:1 v/v) and measuring the background lysis in the absence of peptide. For 100% hemolysis, the erythrocytes were added to 0.2% of Triton X-100 (Sigma, Germany) in the same buffer, giving a final concentration of 0.1% Triton X-100. Incubation was performed at 37°C for 20 min with gentle shaking. The tubes were centrifuged at 20 000 g for 5 min to pellet the cells, and the absorbance at 540 nm was recorded against water. The percentage lysis was then calculated relative to 0% lysis with buffer and 100% lysis by Triton X-100. The absorbance measurement was repeated three times, and the averaged values are used.

### Cell Uptake Assay

HeLa TK A549 cells (from DSMZ, Braunschweig, Germany) were cultivated in minimal essential medium (MEM) with 10% fetal calf serum (FCS) (PAA, Cöble, Germany), and Glutamax (Invitrogen, Karlsruhe, Germany), without antibiotics. Cell density after incubation over night was 30000 cells/chamber in 300 µL medium. For experiments, the medium was removed and cells incubated with peptides at the respective concentration in HEPES Krebs Ringer (HKR) buffer, which consists of 5 mM HEPES, 137 mM NaCl, 2.68 mM KCl, 2.05 mM MgCl_2_, 1.8 mM CaCl_2_, and 5.55 mM glucose, pH 7.4. After 30 min (37°C, 5% CO_2_) cells were washed with HKR buffer, and the behavior of cells at variable peptide concentration examined. A confocal microscope type SP 5 from Leica was used for fluorescence microscopy. The 488 nm line of an argon laser was used for excitation of calcein and the 633 nm line of a helium-neon laser was used for excitation of Fluka 630 red. For kinetic experiments, cells were washed after incubation for 30 minutes and placed into MEM medium at 37°C, and studied after 1, 2, 4, 8 and 24 h. Five minutes before microscopy, calcein AM (Fluka, Buchs, Switzerland) was added to a final concentration of 1 nM to examine cell viability. To avoid artifacts, cells were not fixed.

### Vesicle Fusion Assay

The extent of peptide-induced lipid vesicle fusion was measured using a lipid mixing assay based on Förster Resonance Energy Transfer (FRET) between NBD- and rhodamine-labeled lipids, as previously described in detail (Reichert et al., 2007; Wadhwani et al., 2012b). Vesicles containing both dyes at 2 mol-% each were mixed with dye-free vesicles at a 1:10 molar ratio to give a final lipid concentration of 100 µM in buffer with 150 mM NaCl and 10 mM HEPES at pH 7.5. The initial fluorescence (λ_exc_ = 450 nm, λ_em_ = 530 nm) of the labeled/unlabeled vesicle suspension was taken as 0% lipid mixing, and the extent of mixing was determined by the fluorescence level at 30 min after addition of the peptides (4 µM, P:L = 1:25). The maximum of 100% fusion was defined by the level obtained upon addition of the detergent Triton-X100 to a final concentration of 0.5 mol-%. Experiments were performed at a temperature of 37°C.

Several representative lipid mixtures were used: POPC/POPG (molar ratio 4:1), POPC/CHOL (7:3), POPC/POPG/CHOL (8:2:5), POPE/POPG (7:3), and POPC/POPE/POPS/SM/CHOL (10:5:2:2:10). The fusion activities of BP100, TAT, MAG2, PGLa, MSI-103, and MAP were compared with that of the well-known fusion peptide FP23 from HIV-1 (Freed et al., 1990; Reichert et al., 2007).

### Vesicle Leakage Assay

The peptide-induced lipid vesicle leakage was determined using a fluorescence-based assay as described previously in detail (Grau-Campistany et al., 2015). Leakage of ANTS/DPX from DMoPC/DMoPG (1:1), POPC/POPG (1:1), DErPC/DErPG (1:1), or POPE/POPG (1:1) vesicles was determined at a range of peptide concentration. Vesicle solution was added to a peptide solution in buffer at pH = 7.5, and the fluorescence signal was followed for 10 minutes. The data was scaled to the signal after addition of Triton-X, which gave the value for 100% leakage. Measurements were repeated at least three times.

## Results

### Antimicrobial Activity

We here used three bacterial strains to compare the antimicrobial activity of BP100 with those of other well-known AMPs and CPPs. **Table 2** shows that the cell penetrating peptide TAT has a very low antimicrobial activity (MIC was at least 125 µM), whereas MAP – which is also nominally a CPP – was even more active than the designated AMPs. Against *E. coli*, BP100 is the most active peptide, against *E. helveticus* and *K. rhizophila* MSI-103 and MAP are slightly better than BP100. In a previous study (Wadhwani et al., 2012a), MIC values of the same peptides were determined against two Gram-negative strains (*E. coli* DSM 1103 and *Pseudomonas aeruginosa* DSM 1117) and two Gram-positive strains (*Staphylococcus aureus* DSM 1104, and *Staphylococcus epidermidis* DSM 1798). These values are included in **Table 2** for comparison. Here, MSI-103 was the most active peptide, followed by BP100 and MAP. PGLa had a lower activity and MAG2 was even less active. TAT had essentially no activity against the tested bacteria.

**Table 2:** Minimum inhibitory concentration of the investigated peptides against tested bacteria, hemolytic activity, and toxicity against HeLa cells (all values in μM).

Peptide	MIC^a^			MIC^b^				HC_10_ ^c^	HeLa cells^d^
	*E. coli* (DSM 498)	*E. helveticus*	*K. rhizophila*	*E. coli* (DSM 1103)	*P. aeruginosa*	*S. aureus*	*S. epidermidis*		
BP100	6.3	6.3	3.2	10	20	5	5	45	4
MAG2	75	12.5	12.5	99	>99^e^	99	99	>100	20
PGLa	25	16.3	4.1	30	119	15	7	16	10
MSI-103	12.5	1.6	0.8	3	28	3	2	9	4
MAP	25	3.2	0.4	31	>122^e^	8	4	2	2
TAT	>500^e^	500	125	>128^e^	128	128	>128^e^	>>100	> 20^e^

a New results from this study.

b MIC values from (Wadhwani et al., 2012a).

c Concentration that induced 10% hemolysis.

d Lowest tested concentration where dead HeLa cells were observed.

e No effect was found for the highest tested concentration.

### Hemolysis

**Figure 2:**
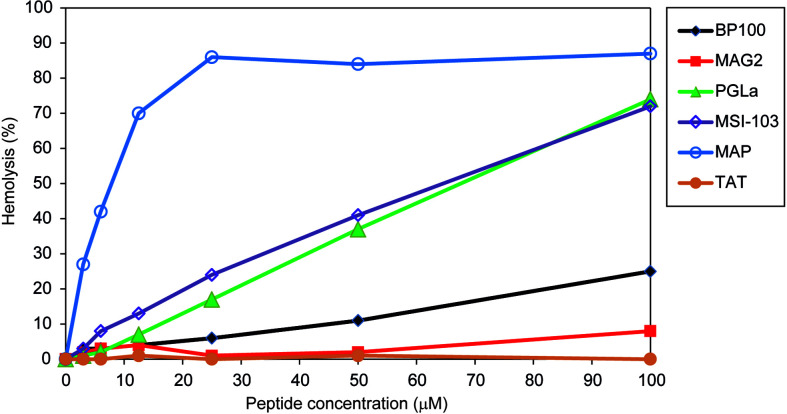
Hemolysis of the different peptides. BP100 has a low hemolytic activity. The longer α-helical peptides have very different effects, from low for MAG2 to very high for MAP, whereas TAT gives hardly any hemolysis in the tested concentration range.

BP100 had also been optimized to have low hemolytic side-effects (Badosa et al., 2007). We thus compared the hemolysis of BP100 with that of other AMPs and CPPs. Hemolysis was measured at peptide concentrations up to 100 µM. These data are summarized in **Table 2**, giving the concentration of each peptide needed to induce 10% hemolysis, and complete graphs are shown in **Figure 2**. BP100 has a lower activity than the other AMPs, except for MAG2, and also lower than the nominal CPP MAP. The highly charged CPP TAT, on the other hand, has hardly any hemolytic activity. In this assay, BP100 resembles the long pore forming amphipathic helical peptides, especially when the data are re-scaled according to the different weights of peptides rather than to their molar concentrations.

### Cell Uptake

**Figure 3:**
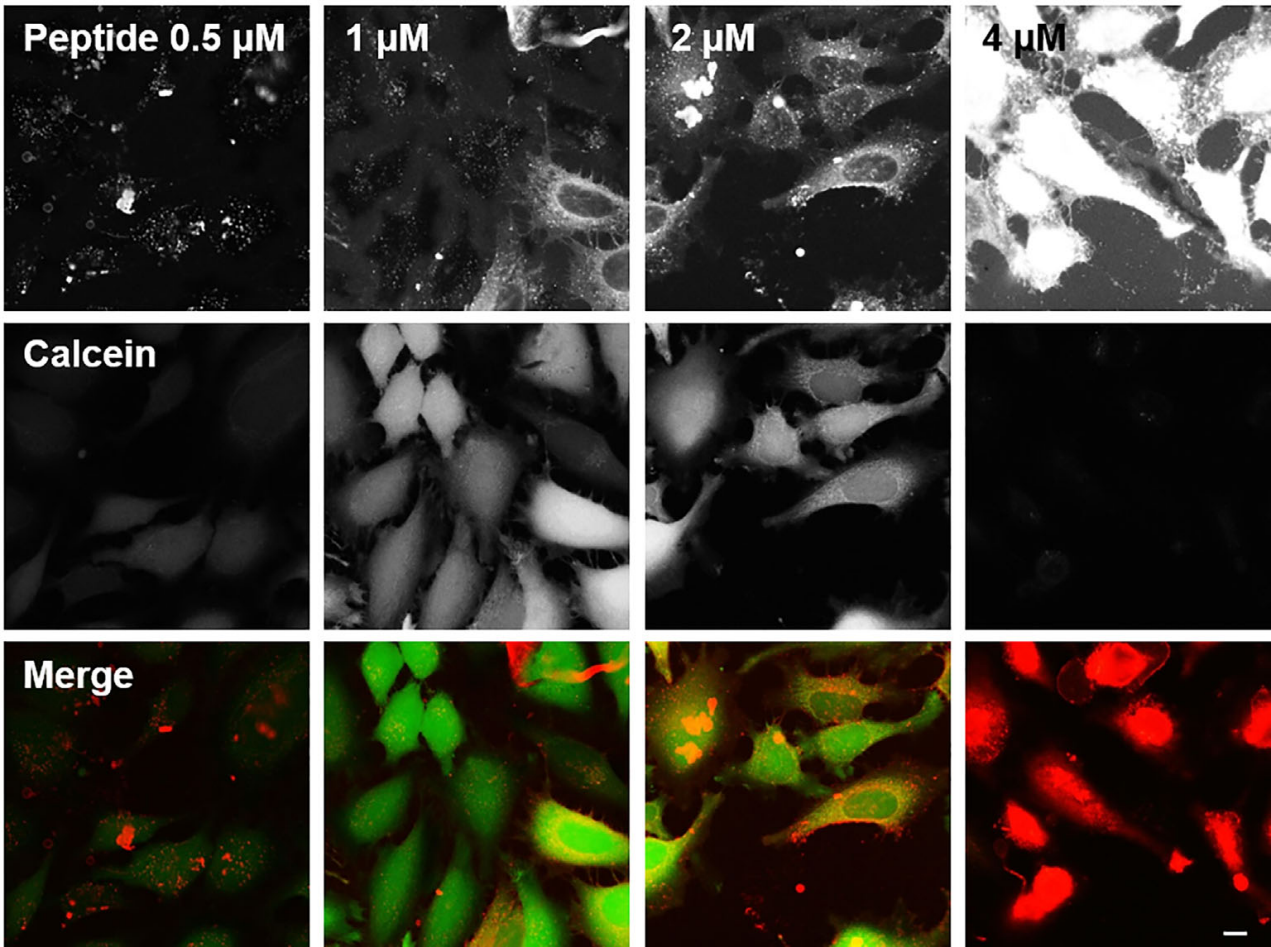
Cell uptake of BP100 in HeLa cells. The top row shows the signal of the peptide labeled with Fluka 630 red (displayed as red channel in the merged images at the bottom). The middle row shows the signal from calcein AM, which emits a green fluorescence when taken up by living cells (displayed as green channel in the merged images). The bottom row shows the superposition of the two channels: Overlapping red and green signals give a yellow color where BP100 has entered a living cell, while red images show cells that are permeabilized and dead. For each column the concentration of BP100 is given in the top row. Scale bar 25 µm.

A cell uptake assay was performed in human HeLa cells, and the uptake of BP100 was compared with that of the other AMPs and CPPs. Cell uptake was studied by fluorescence microscopy, using peptides labeled at the N-terminus with Fluka 630 Red. In **Figure 3**, the results are shown for cells incubated with BP100 at concentrations of 0.5, 1, 2 or 4 µM. Cells were also incubated with calcein AM, which is taken up by healthy living cells and cleaved by esterases to give a green fluorescent signal in contact with intracellular calcium. When the peptide and calcein signals overlap, a yellow signal appears. In **Figure 3**, the signals from the separate channels are shown together with the superimposed signals from both channels. The punctuate patterns of the peptides inside cells thus suggest that BP100 is taken up by endocytosis, with a good uptake at 2 µM. However, at 4 µM BP100 begins to show considerable toxicity against HeLa cells as seen by an increased number of rounded cells.

The natural AMPs MAG2 (see **Supporting Figure S1**) and PGLa (**Figure S2**) showed less uptake into HeLa cells than BP100, yet they also entered by endocytosis (i.e. they did not truly penetrate across the plasma membrane). These peptides were less toxic, with dead cells found at 20 µM for MAG2, and 10 µM for PGLa. The designer-made AMP MSI-103 (**Figure S3**) showed endocytotic cell uptake with a similar efficiency as PGLa and MAG2, but its cytotoxic concentration was similar to BP100. The designer-made CPP MAP (**Figure S4**) was highly toxic already at 2 µM, and therefore cell uptake at higher concentrations could not be investigated. On the other hand, TAT (**Figure S5**) showed excellent cell uptake, with no sign of toxicity even at 20 µM. Also in this case a punctuate pattern indicates uptake by endocytosis. The unstructured TAT peptide is indeed a widely used CPP, and it enters the cells in a more efficient manner than the amphipathic helical peptides, which also seem to be more toxic. In this CPP assay, the behavior of BP100 clearly resembles that of the magainin family of pore forming peptides more than that of TAT.

Useful CPPs should have a negligible toxic effect, so the toxicity of the tested peptides is compared in **Table 2**. In HeLa cells, the numerical toxic value was taken as the lowest concentration where dead cells were observed. In erythrocytes, we defined the toxic threshold as the peptide concentration that gave 10% hemolysis. The lowest overall toxicity was found for the highly charged TAT peptide, followed by MAG2 and PGLa. BP100 was not very toxic against erythrocytes, but turned out to be one of the most toxic peptides against HeLa cells. It can be noted that the most toxic peptides against both HeLa cells and erythrocytes were MAP and MSI-103, two designer-made sequences. Again, we find considerable similarity between the short BP100 and the longer helical peptides, but little resemblance to TAT.

### Vesicle Fusion Activity

**Figure 4:**
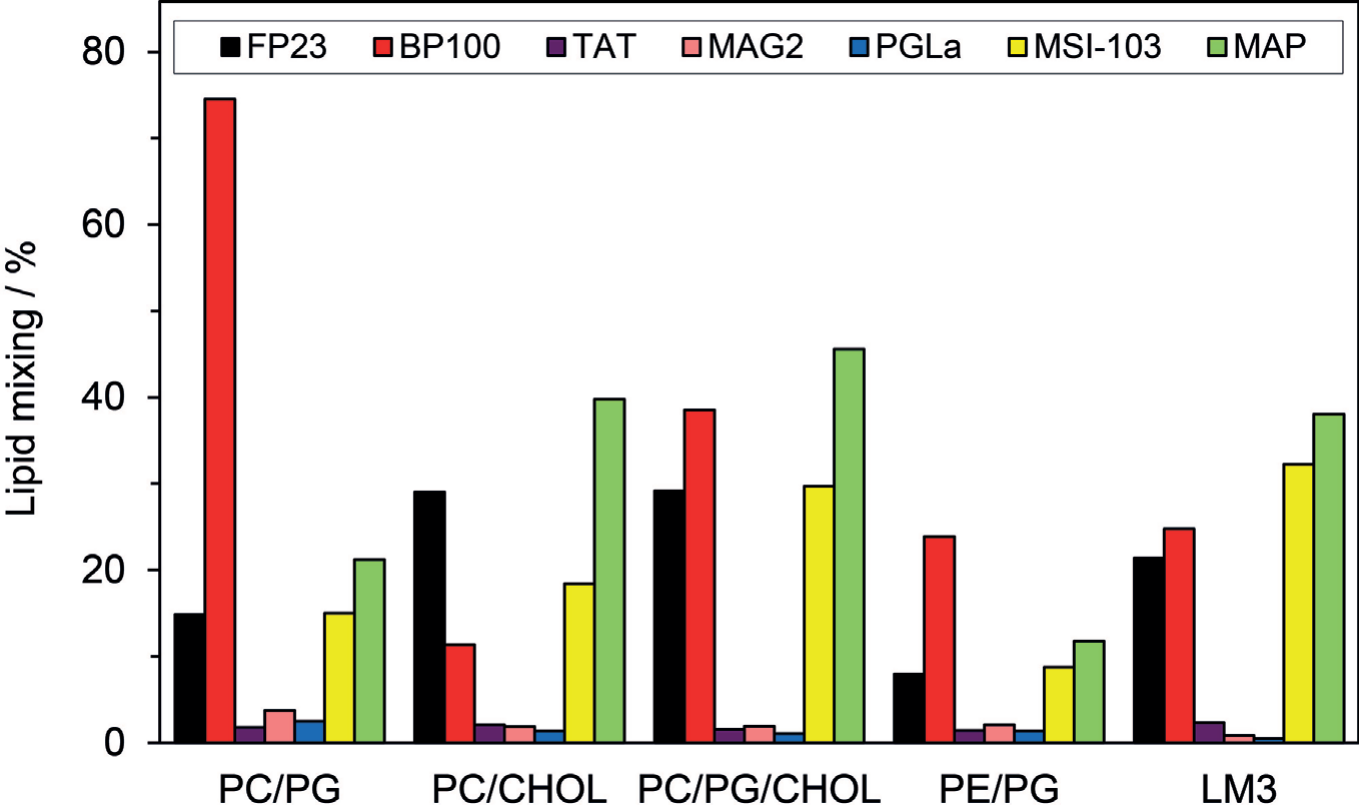
Extent of lipid vesicle fusion induced by BP100, HIV-TAT, and the long amphiphilic helical peptides PGLa, MAG2, MSI-103 and MAP; the fusion peptide FP23 from HIV-1 is used as a control. Vesicles with the following lipid compositions were used: POPC/POPG (molar ratio 4:1), POPC/CHOL (7:3), POPC/POPG/CHOL (8:2:5), POPE/POPG (7:3), and POPC/POPE/POPS/SM/CHOL (10:5:2:2:10) which is called LM3. The P/L molar ratio was 1:25. Peptide-induced fusion was measured by a lipid mixing assay based on Förster resonance energy transfer (FRET) between NBD-PE and Rh-PE as described in the Methods section.

Typical so-called fusion peptides, e.g. from viruses, can induce fusion of membranes, and even some AMPs and CPPs are able to do so. To compare the fusogenic activity of BP100 and the other peptides, we carried out a standard fluorescence lipid mixing assay (Wadhwani et al., 2012b) for several representative lipid compositions. The extent of peptide-induced fusion was examined in small unilamellar vesicles consisting of POPC/POPG (molar ratio 4:1), POPC/CHOL (7:3), POPC/POPG/CHOL (8:2:5), POPE/POPG (7:3), and POPC/POPE/POPS/SM/CHOL (10:5:2:2:10). The latter composition is also called “LM3” and is often used as a model membrane in viral fusion assays, mimicking the lipid head groups and cholesterol content of a typical eukaryotic cell (Aloia et al., 1993; Yang et al., 2003). The fusion activity of the tested peptides was compared with that of the well-known fusion peptide FP23 from HIV-1. The results are shown in **Figure 4**. In all vesicles containing anionic lipids, BP100 (charge: +6) induced more fusion than FP23 (charge: +2), indicating that BP100 is a highly effective fusogenic agent. This finding suggests that initial electrostatic attraction is important for membrane fusion. In uncharged vesicles of POPC/CHOL, on the other hand, BP100 gives less fusion than FP23, in line with poor binding of the more water-soluble BP100. The highly charged TAT (+8) gives essentially no fusion in any lipid system. Interestingly, the two natural AMPs from frog skin, MAG2 and PGLa, have very low fusion activities in all tested lipid systems. On the other hand, the two related magainin-derived designer-helices MSI-103 and MAP give strong fusion, comparable to BP100.

### Vesicle Leakage Activity

The ability of BP100 and the other peptides to induce leakage in POPC/POPG (1:1) vesicles was studied as a function of peptide concentration using a fluorescence assay. The results in **Table 3** show that the peptides exhibit very different activities. TAT induces basically no leakage, even at a very high P/L of 1:12.5. BP100 gives less leakage than the longer helical peptides. For example, at 1:50 where the latter peptides give almost 100% leakage, BP100 gives only 26%. Of the longer helical peptides, MSI-103 is the least active and MAP the most active; PGLa and MAG2 show similar activity.

**Table 3:** Vesicle leakage (in %) induced by the tested peptides in POPC/POPG (1:1) vesicles at different P/L values.

Peptide	P/L					
	1:400	1:200	1:100	1:50	1:25	1:12.5
BP100	- ^b^	9 ± 3	15 ± 4	26 ± 3	51 ± 3	100
MAG2	27 ± 9	83 ± 11	96 ± 5	100	100^c^	100^c^
PGLa	24 ± 2	80 ± 3	100	100	100^c^	100^c^
MSI-103	- ^b^	16 ± 4	52 ± 8	83 ± 3	100	100^c^
MAP	52 ± 1	100	100	100	100^c^	100^c^
TAT	- ^b^	- ^b^	- ^b^	4	6 ± 3	5

a Rounded to whole numbers. When all repetitions gave the same value, the standard deviation is zero and is not given.

b Not measured.

c Assumed to be 100% since this was found at a lower concentration. The mean value and standard deviation from 3-5 measurements are given.

An earlier leakage study had been carried out systematically on a series of so-called KIA peptides, which are composed of the same repetitive KIAGKIA-motif as MSI-103 but differ incrementally in length. This study had demonstrated that peptide-induced vesicle leakage was critically dependent on hydrophobic matching, i.e. on the relative length of the helix compared to the thickness of the lipid bilayer (Grau-Campistany et al., 2015). Therefore, we performed some further leakage experiments here on BP100 and MSI-103 in several other lipid systems with different bilayer thickness (see **Supporting Information Table S1**). In thin membranes of DMoPC/DMoPG (1:1) both peptides gave 100% leakage even at 1:100, and in thick membranes of DErPC/DErPG (1:1) both peptides gave essentially no leakage. In POPE/POPG (1:1), both peptides gave lower leakage than in POPC/POPG (1:1).

## Discussion

In this study, we have used biological and biophysical assays to compare the membrane active peptide BP100 with several AMPs and CPPs, that are generally considered to represent distinctly different mechanisms of action. Our goal is to better understand the versatile mode of action of BP100, a very short multifunctional peptide.

We thus present a comparison of BP100 with PGLa, MAG2, MSI-103, MAP and TAT. As shown by CD previously, BP100 folds into a compact amphiphilic α-helix with relatively high helix content when bound to a lipid membrane (Wadhwani et al., 2014; Zamora-Carreras et al., 2016), and the binding is predominantly electrostatic. It is also known from earlier CD analyses that PGLa (Matsuzaki et al., 1998; Glaser et al., 2004), MAG2 (Matsuzaki et al., 1998), MSI-103 (Blazyk et al., 2001; Strandberg et al., 2008) and MAP (Wadhwani et al., 2008) form amphiphilic α-helices in a lipid environment. However, these latter membranolytic peptides with 18-23 amino acids are long enough to span a typical membrane in a transmembrane orientation. BP100, composed of only 11 amino acids, is too short to span the membrane, and yet it shows pronounced antimicrobial activity. TAT, on the other hand, is just as short as BP100 and carries even more charges. But in contrast to BP100, TAT shows a random coil CD spectrum both in aqueous solution (Futaki et al., 2001) and in the presence of lipid vesicles (Wadhwani et al., 2012a).

**Figure 5:**
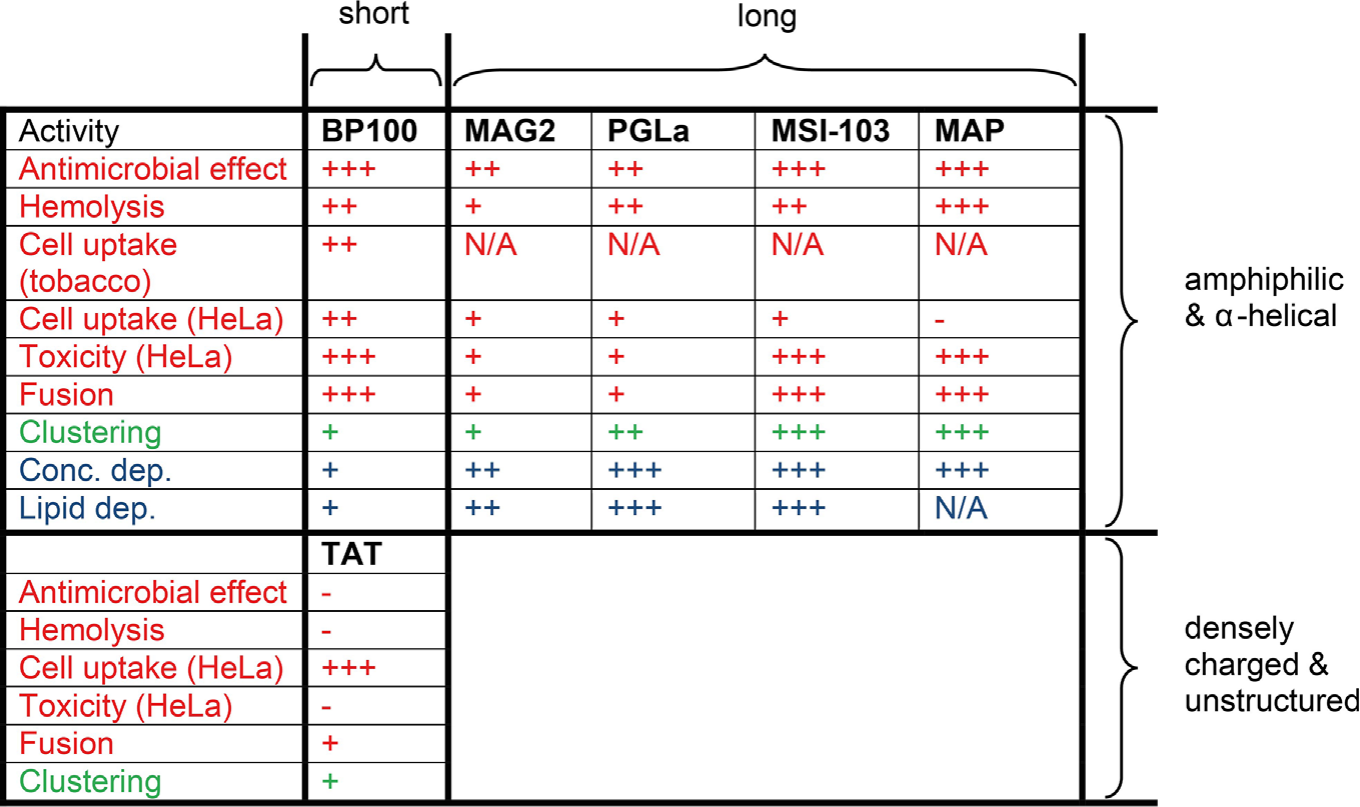
Overview of all available results on the different membrane-active peptides. Short peptides contain only 11-14 amino acids, while the long ones consist of 18-23 amino acids. The newly measured activities are shown in red; previously reported lipid clustering is indicated in green [from ref. (Wadhwani et al., 2012a)]; and in blue we state whether any structural changes are known to take place as a function of peptide concentration or lipid composition, according to previous NMR and OCD analyses. The effects are coded as: (-) negligible effect, (+) low effect, (++) medium effect, (+++) strong effect, (N/A) no data available.

An overview of the different peptides and their activities is shown in **Figure 5**. The question is now whether (i) BP100 behaves similar to the long α-helical peptides, or whether (ii) it rather resembles the short, highly charged TAT, or whether (iii) it shows an altogether different behavior? It has been clearly demonstrated that the longer peptides can assemble into oligomeric transmembrane pores of the toroidal wormhole type (Zerweck et al., 2017; Strandberg et al., 2020b). The most critical requirement for pore formation in different types of membranes is that the helix must have a sufficient length to span the thickness of the relevant lipid bilayer under investigation (Grau-Campistany et al., 2015; Grau-Campistany et al., 2016). This concept of hydrophobic matching, combined with the architecture of an oligomeric peptide pore lined with some lipid head groups, can explain their mechanism of action very well. However, this mode of action is not possible for the BP100 peptide, because it is far too short. It thus seems more likely that the compact BP100 helix disturbs the membrane in other ways and makes it more permeable, for example by the “carpet mechanism” (Oren and Shai, 1998). From the lipid mixing assay, we note that BP100 is highly fusogenic, presumably by temporarily exposing hydrophobic lipid segments upon binding, which may also contribute to its mechanism of action.

The biological activity of the different peptides has been thoroughly studied, both here and in the literature. But it is hard to compare data from different studies; to compare the biological activity the peptides should be tested under identical conditions using the same bacteria, same batch of blood, same preparation of vesicles etc. Therefore, we here present new experiments on the full series of peptides. Here, we found that BP100 is the most active antimicrobial peptide against the tested bacteria, together with MSI-103. MAP is also very active, while PGLa and MAG2 show intermediate, and TAT almost no antimicrobial effect (**Table 2**). These results are compatible with a previous study of these and other peptides in other bacteria (Wadhwani et al., 2012a), where the order of activity was found to be MSI-103 > BP100 > MAP > PGLa > MAG2 >> TAT. Thus, with respect to antimicrobial activity, BP100 has more in common with the longer α-helical AMPs, and does not behave like TAT. We can note that BP100 shows a very similar MIC towards the tested Gram-positive and Gram-negative bacteria, an observation that has also been previously reported for its alanine mutants (Zamora-Carreras et al., 2016). This behavior is in contrast with the other AMPs and MAP, which are 6-60 times less active against Gram-negative *E. coli* than against Gram-positive *K. rhizophila* (**Table 2**).

With regard to hemolysis, BP100 has a significantly lower hemolytic activity than the longer α-helical peptides MAP, PGLa and MSI-103, but still higher than MAG2, whereas TAT shows almost no activity (**Figure 2**). These results fits with a previous study, where hemolytic activity was also reported in the order MAP >> MSI-103 ≈ PGLa (Strandberg et al., 2007). TAT previously did not show any hemolytic activity (Sabatier et al., 1991; Wang et al., 2010). Considering that **Table 2** (and **Figure 2**) gives the percent of hemolysis as a function of concentration in µM, it can be noted that BP100 contains only half as many amino acids as PGLa and MSI-103. Therefore, by rescaling these data to the same mass of peptide (to have a comparable membrane surface coverage for all peptides) we would get rather similar activities for BP100, PGLa and MSI-103 (**Table 2**, given that the hemolysis increases approximately linearly with concentration). It is apparent that the different α-helical amphipathic peptides do not induce the same degree of hemolysis, as MAP is much more active than PGLa and MAG2 much less, in spite of their similar length and secondary structure. On the other hand, it is very clear that TAT has almost no hemolytic effect. Therefore, BP100 certainly does not behave like TAT regarding hemolysis.

Another kind of action of membrane-bound peptides is to facilitate fusion between membranes of vesicles or cells. We find here that BP100 has strong fusogenic activity (**Figure 4**), even higher than the dedicated fusion peptide, FP23 from HIV-1, except in uncharged POPC/CHOL. TAT shows very little fusion in all tested lipid systems. PGLa and MAG2 also showed a very small fusogenic effect, while MAP and especially MSI-103 had a pronounced effect, considerably stronger than FP23. These fusion results fit well with a previous comparison of different AMPs and CPPs (Wadhwani et al., 2012b). Obviously, the fusogenic effect is very different even between amphipathic α-helical peptides of similar length. It can be noted, however, that fusion is generally very strong for the designed peptides (MAP, MSI-103, BP100) which were optimized for other activities (high antimicrobial and low hemolytic activity). In contrast, the natural AMPs (PGLa and MAG2) seem to have been optimized by evolution to avoid any such kinds of side-effects. TAT had not yet been tested in the previous study (Wadhwani et al., 2012b), but a mutant called TATP59W (sequence Ac-GRKKRRQRRRPWQ-NH_2_) was used in another report, and a high extent of lipid mixing was found (Thorén et al., 2005). Fusion results from different studies are ambiguous to compare quantitatively, because experiments were not done in an identical way and vesicles of different lipid compositions were used. Yet, we can note that BP100 and the longer helical AMPs have a distinct fusogenic activity, whereas TAT is inactive.

Vesicle leakage was measured here for BP100 in the same lipid vesicle systems as previously used for MSI-103 and related peptides (Grau-Campistany et al., 2015; Gagnon et al., 2017). BP100 showed less leakage in POPC/POPG (1:1) vesicles than the longer α-helical peptides (**Table 3**), at the same peptide-to-lipid molar ratio. Recalculated as mass ratios, the difference is smaller. The leakage of BP100 is most similar to that of MSI-103. At a high P/L of 1:12.5, also BP100 gives full leakage. In contrast, TAT gave essentially no leakage at all, even at a very high peptide concentration. Also in this respect, BP100 behaves more like the other long helical peptides than like TAT.

For the KIA series of peptides a threshold length for leakage was found, corresponding to the hydrophobic thickness of the membrane. For thin membranes of DMoPC/DMoPG, KIA peptides with 14 amino acids gave leakage, in medium POPC/POPG 17 amino acids were needed, and in thick DErPC/DErPG vesicles, the shortest peptide giving leakage had 24 amino acids. The length of α-helices with these number of residues fit very well with the corresponding hydrophobic thickness of the membranes (Grau-Campistany et al., 2015). Interestingly, we found here that BP100 – even though it is nominally too short with an estimated length of 16.5 Å – induced full leakage in DMoPC/DMoPG (with a hydrophobic thickness (d_H_) of 19.2 Å, (Marsh, 2008)) and POPC/POPG (d_H_ = 27-28 Å, (Kucerka et al., 2006; Pan et al., 2012)) at P/L=1:12.5, but no leakage in DErPC/DErPG (d_H_ = 34.4 Å, (Kucerka et al., 2006)). Obviously, it seems to be easier to induce leakage through a thinner membrane, irrespective of the type of mechanism, whether pore formation or any other kind of action. Comparing the activity of BP100 with that of KIA peptides, BP100 falls in between KIA15 and KIA17 (Grau-Campistany et al., 2015). This comparison shows that BP100 induces more leakage than would be expected for its length of 11 amino acids, provided that BP100 was to operate by the same mechanism of action as the KIA peptides.

BP100 is also a remarkable CPP that has been previously shown to be taken up in tobacco cells (Eggenberger et al., 2011; Gao et al., 2016; Eggenberger et al., 2017). Here, we observed also some good uptake into HeLa cells, most likely *via* endocytosis. However, already at 4 µM BP100 was toxic and caused cell death (**Figure 3**). This finding clearly indicates that BP100 is unsuitable as a CPP for these HeLa cells, in contrast to its successful CPP applications in tobacco cells (Eggenberger et al., 2011; Gao et al., 2016; Eggenberger et al., 2017). The longer α-helical peptides showed varying effects. PGLa and MAG2 elicited a lower degree of presumably endocytotic uptake, accompanied also by less toxicity (**Figures S1** and **S2**). MSI-103 showed good endocytotic uptake but was toxic already at 4 µM (**Figure S3**), while MAP was toxic already at 2 µM with no uptake at any lower concentrations (**Figure S4**). We can thus note here (as for fusion) that all designed α-helical peptides (BP100, MSI-103, MAP) caused significant toxic side-effects, while the natural peptides (PGLa and MAG2) were less toxic. TAT belongs to yet another category, showing excellent uptake, probably *via* endocytosis, and no toxicity (**Figure S5**). We can thus conclude that BP100 does not behave like TAT regarding cell uptake and toxicity. The results are in line with a previous study, where the uptake of MAG2 and TAT[47-57] in HeLa cells showed that MAG2 was taken up more efficiently, yet with higher toxicity, which was attributed to membrane pore formation (Takeshima et al., 2003).

In an earlier study of lipid clustering induced by various representative membrane-active peptides (Wadhwani et al., 2012a), it was shown that BP100, MAG2 and TAT had a low lipid clustering effect, while PGLa, MSI-103 and MAP showed a high ability to segregate anionic lipids. The low activity of MAG2 can be readily attributed to its low charge. However, despite the high number of charges on BP100 and TAT, it seems that their short length and high dynamics make it difficult for these two peptides to cluster anionic lipids. This scenario is one case where BP100 actually behaves similarly to TAT.

BP100 is known to form an amphiphilic α-helix when bound to a membrane, just like the longer peptides compared here. However, our earlier solid-state NMR and oriented CD studies showed a fundamentally different type of behavior for BP100 than for the other, longer amphipathic α-helices. When using OCD to examine the re-alignment of the membrane-bound helices as a function of peptide concentration, we found that PGLa, MSI-103 and MAP are oriented flat on the membrane surface. This is the preferred state at low concentration in DMPC bilayers, but above a certain threshold concentration, P/L*, the helices flip into a tilted state, which allows them to insert partly into the membrane at an oblique angle (Bürck et al., 2008). P/L* varies from 1:236 for MSI-103 (the most antimicrobially active of the peptides) to 1:85 for PGLa (Bürck et al., 2008). A similar behavior had been reported for MAG2 (Ludtke et al., 1994). The importance of P/L* for the mechanism of action of AMPs has been a matter of debate in the literature (Ferre et al., 2009; Melo et al., 2009). Solid-state ^15^N-, ^19^F- and ^2^H-NMR revealed further structural details of the helix re-orientation in membranes for PGLa (Glaser et al., 2005; Strandberg et al., 2006; Tremouilhac et al., 2006), MAP (Wadhwani et al., 2008), MSI-103 (Strandberg et al., 2008; Strandberg et al., 2012; Strandberg et al., 2020b), and KIA peptides (Grau-Campistany et al., 2016). These peptides all change their orientation as a function of peptide concentration and membrane composition.

In contrast to these long helices undergoing a distinct re-orientation, BP100 did not show any change in alignment for P/L ratios between 1:3000 and 1:10 in our earlier ^19^F-NMR analysis (Wadhwani et al., 2014). In this respect, BP100 is more similar to MAG2, which also does not change its orientation much as a function of peptide concentration and membrane composition (Strandberg et al., 2016). A possible reason for the difference between MAG2 and for example PGLa and MSI-103 is that MAG2 has a charged C-terminus, whereas PGLa and MSI-103 are amidated at the C-terminus, and it was shown for KIA peptides that a charge at the C-terminus makes it much harder for a peptide to insert into the membrane and induce leakage (Strandberg et al., 2020a). BP100 is also amidated on the C-terminus so there must be some other reason for its lower propensity to change its orientation. A concentration dependent flip of the helix tilt angle is very likely a sign of dimerization or assembly into higher oligomers. This seems to be an important step in the pore forming mechanism of the longer α-helical peptides, but not in the case of BP100.

OCD and solid-state NMR data are easy to analyze for α-helical peptides, but unfortunately these experiments are not meaningful for unstructured arginine-rich peptides like TAT. Given that the interaction of these charged side chains with the phosphate head groups of the lipids is well documented (Frigyes et al., 2001), the TAT-like peptides are expected to remain bound to the membrane surface, below the threshold concentration at which they can transverse the bilayer.

We have previously demonstrated that the lipid composition can have a dramatic effect on the membrane alignment of MSI-103 and PGLa (Strandberg et al., 2012; Strandberg et al., 2013). For BP100, solid-state NMR showed no clear difference between different lipid systems. Only in the presence of lyso-lipids a slightly more tilted orientation was found for BP100 and also for other peptides (Wadhwani et al., 2014; Misiewicz et al., 2015; Zamora-Carreras et al., 2016). Thus, it seems that the orientation of the short and compact BP100 helix is far less influenced by the membrane properties than the orientation of the longer α-helical AMPs. The helix axis of BP100 is essentially always found to be flat on the bilayer as a time-average, but NMR showed that BP100 executes some highly dynamic rigid-body wobbling in liquid crystalline membranes.

To summarize the known structural features, the short amphiphilic α-helical BP100 does not behave in the same way as the longer α-helical peptides in membranes. In particular, it does not show any signs of self-assembly and exhibits only a very weak lipid dependence. Instead, it always remains bound to the membrane surface as highly mobile monomers, according to solid-state NMR and OCD. On the other hand, our present biological assays show a functional behavior for BP100 that is very similar to the long amphiphilic α-helices of MSI-103 and MAP, all of which are designer-made. Compared to the natural antimicrobial peptides PGLa and MAG2, BP100 has a higher antimicrobial activity, but also higher side-effects like fusion and toxicity.

Considering the short arginine-rich peptide TAT, it resembles the short lysine-rich BP100 only in its inability to cluster anionic lipids. In all other aspects, BP100 showed very different biological activities, probably due to the pronounced amphipathic character of the BP100 α-helix, which must be responsible for the observed range of quite aggressive membrane activities. Even if both BP100 and TAT are small and dynamic, BP100 forms a rigid helix, in contrast to TAT, which remains flexible and may therefore not disturb the bilayer as much as BP100.

## Conclusions

Magainin-type peptides, which are 18-23 amino acids long and form amphipathic α-helices in the presence of membranes, have strong membrane-perturbing activities. They form pores in membranes and can kill bacteria, induce hemolysis, fusion and leakage of vesicles, and they can even enter cells, though with considerable toxicity due to membrane damage. TAT, on the other hand, which consists of 13 amino acids and is largely unstructured, can enter the cytosol efficiently and has no significant membranolytic effects, i.e., it does not kill bacteria or induce hemolysis as seen in several assays used here. BP100, the interesting hybrid under investigation here, is short with high charge density (like TAT), but folds into an amphipathic α-helix (like the magainin-type peptides). We clearly found that BP100, consisting of only 11 amino acids, behaves rather similarly to the long helical peptides, but very much unlike TAT. This indicate that the mechanism of action of BP100 is rather similar to that of the longer helical peptides. Whereas TAT can cross the membrane without damage, the mode of action of BP100 is to destroy the membrane, leading to leakage, hemolysis and antimicrobial activity. We think the most important factor is that BP100, and the other helical peptides, can form amphipathic structures which binds and disturbs the membrane and leads to permeabilization. This does not prove that BP100 forms pores, and considering its short length it should not be able to span the membrane in a transmembrane pore, but it could still do membrane damage by other mechanisms.

Overall, the spectrum of biological activities of BP100 resembles most closely that of the designer-made peptides MSI-103 and MAP (21 and 18 amino acids, respectively). PGLa and MAG2 (21 and 23 amino acids, respectively) show much less vesicle fusion, and MAG2 is hardly hemolytic, meaning that the two natural peptides are highly active only against bacterial membranes. We suggest that the high specificity of PGLa and MAG2 is the result of a thorough optimization process by evolution, whereas MSI-103, MAP and BP100 are designer-made peptides. Even though they have been optimized by systematic rounds of screening for high antimicrobial activity and low hemolysis, they still show some cytotoxicity and other side-effects such as membrane fusion. For practical applications as an antimicrobial agent or cell-penetrating peptide in medical use it would thus be needed to further optimize BP100 and/or suitable galenic concepts to minimize the toxic side effects.

## Data Availability Statement

The original contributions presented in the study are included in the article/**Supplementary Material**. Further inquiries can be directed to the corresponding author.

## Author Contributions

PW and AU designed the study. CM, PW, ES, MM, JR, IW and MC performed experiments and analyzed data. ES and AU wrote the manuscript. All authors contributed to the article and approved the submitted version.

## Funding

This work was supported financially by the BIF-TM program of the Helmholtz-Gemeinschaft; by the DFG grant INST 121384/58-1 FUGG; and by the DAAD “Portugal - Acções Integradas Luso-Alemãs/DAAD-GRIC” grant D/07/13644.

## Conflict of Interest

The authors declare that the research was conducted in the absence of any commercial or financial relationships that could be construed as a potential conflict of interest.
