# Supplementary material for: Overlapping Properties of the Short Membrane-Active Peptide BP100 With (i) Polycationic TAT and (ii) α-helical Magainin Family Peptides
Source: Front Cell Infect Microbiol. 2021 Apr 26;11:609542. doi: 10.3389/fcimb.2021.609542 (PMC8107365; doi:10.3389/fcimb.2021.609542)
Supplement: Supplementary file 1 [file DataSheet_1.pdf]

SUPPLEMENTARY MATERIAL

Supplementary tables

Table S1. Vesicle leakage (in %) induced by BP100 and MSI-103 in tested lipid systems at different P/L values.

**P/L**	**1:100**	**1:50**	**1:25**	**1:12.5**
**BP100**				
DMoPC/DMoPG (1:1)	100	100	100[a]	100[a]
POPC/POPG (1:1)	16	26	51	100
DErPC/DErPG (1:1)	≈ 0 [b]	≈ 0 [b]	0	0
POPE/POPG (1:1)	≈ 0 [b]	5	9	75
**MSI-103** [c]				
DMoPC/DMoPG (1:1)	100 [d]	100	100	100
POPC/POPG (1:1) [d]	52	83	100	100
DErPC/DErPG (1:1)	≈ 0 [b]	0	3	4
POPE/POPG (1:1)	N/A [e]	16	N/A [e]	100

[a] Assumed to be 100% since this was found at a lower concentration**.**

[b] Not done, since at a higher concentration no or almost no leakage was found.

[c] Data mostly from (Gagnon et al. 2017), where this peptide was called KIA21.

[d] New data

[e] No data available

Supplementary figures

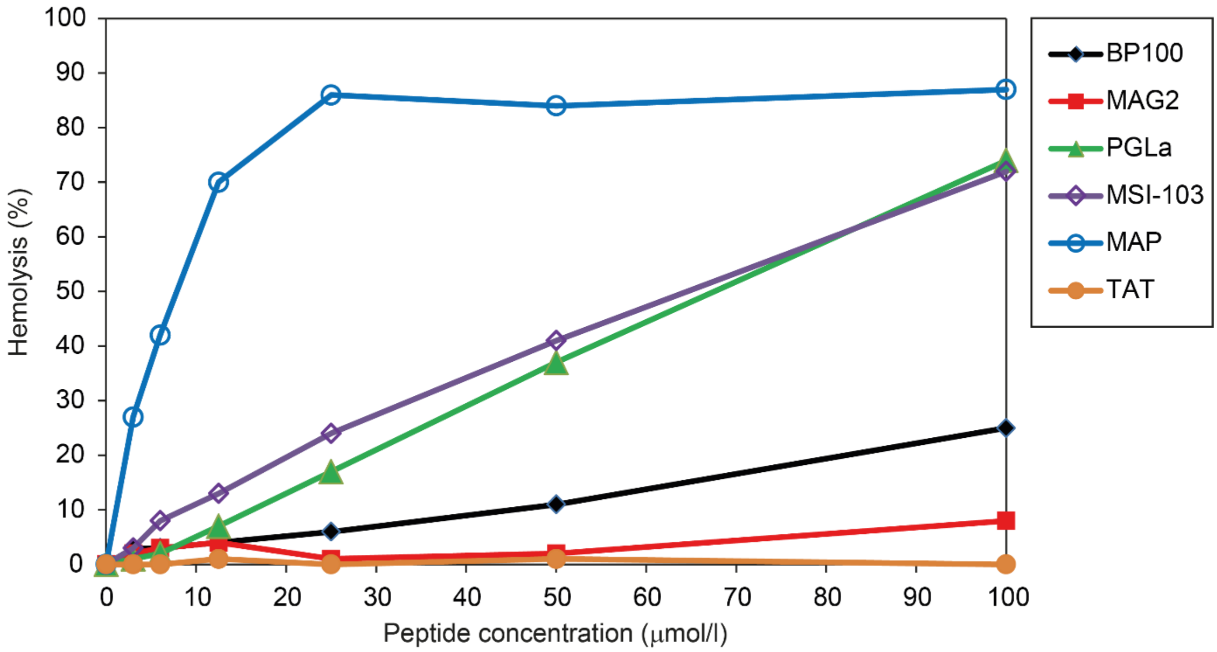

FIGURE S1. Hemolysis of the different peptides. BP100 has a low hemolytic activity. The longer α-helical peptides have very different effects, from low for MAG2 to very high for MAP, whereas TAT gives hardly any hemolysis in the tested concentration range.

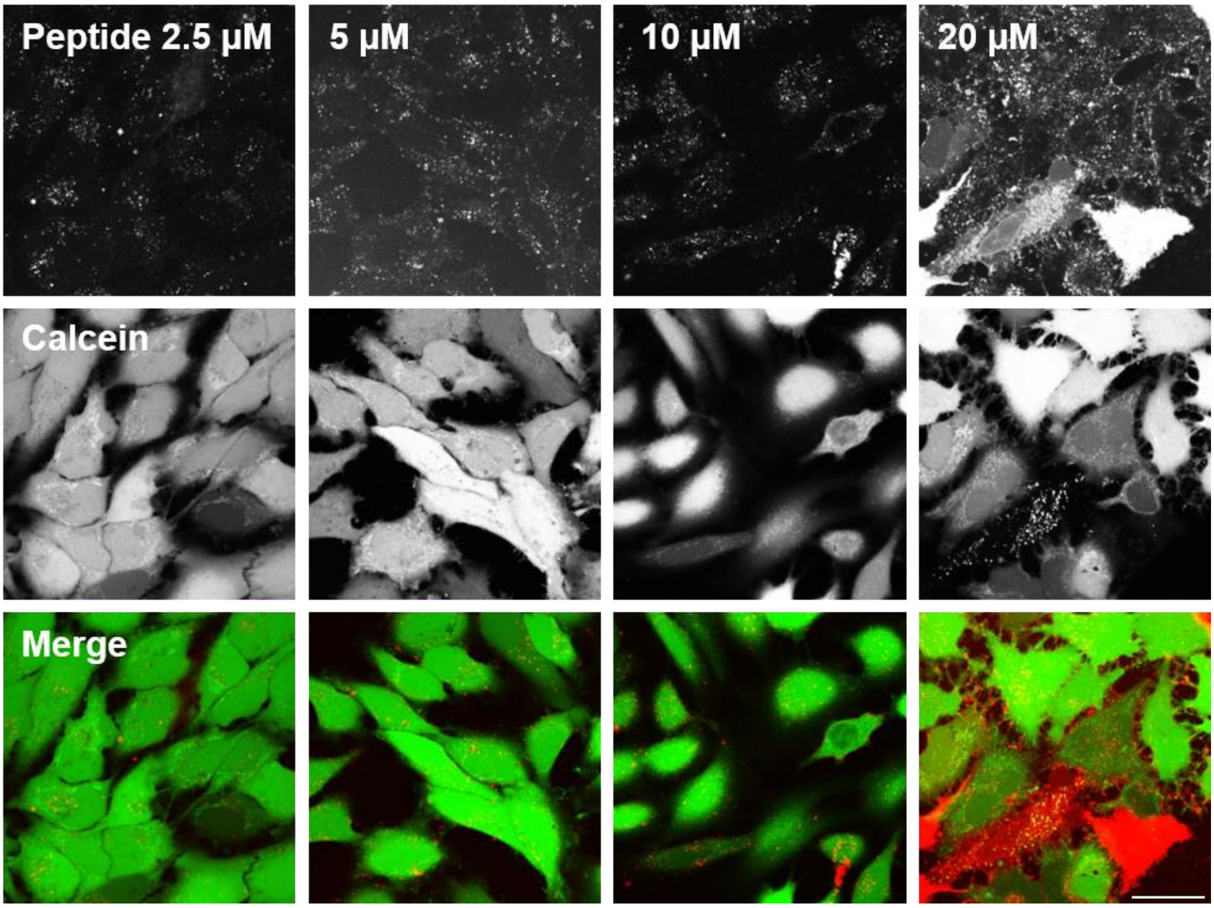

FIGURE S2. Cell uptake of MAG2 in HeLa cells. The top row shows the signal of the peptide labeled with Fluka 630 red (displayed as red channel in the merged images at the bottom). The middle row shows the signal from calcein AM, which emits a green fluorescence when taken up by living cells (displayed as green channel in the merged images). The bottom row shows the superposition of the two channels: Overlapping red and green signals give a yellow color where MAG2 has entered a living cell, while red images show cells that are permeabilized and dead. For each column the concentration of MAG2 is given in the top row. Scale bar 20 µm.

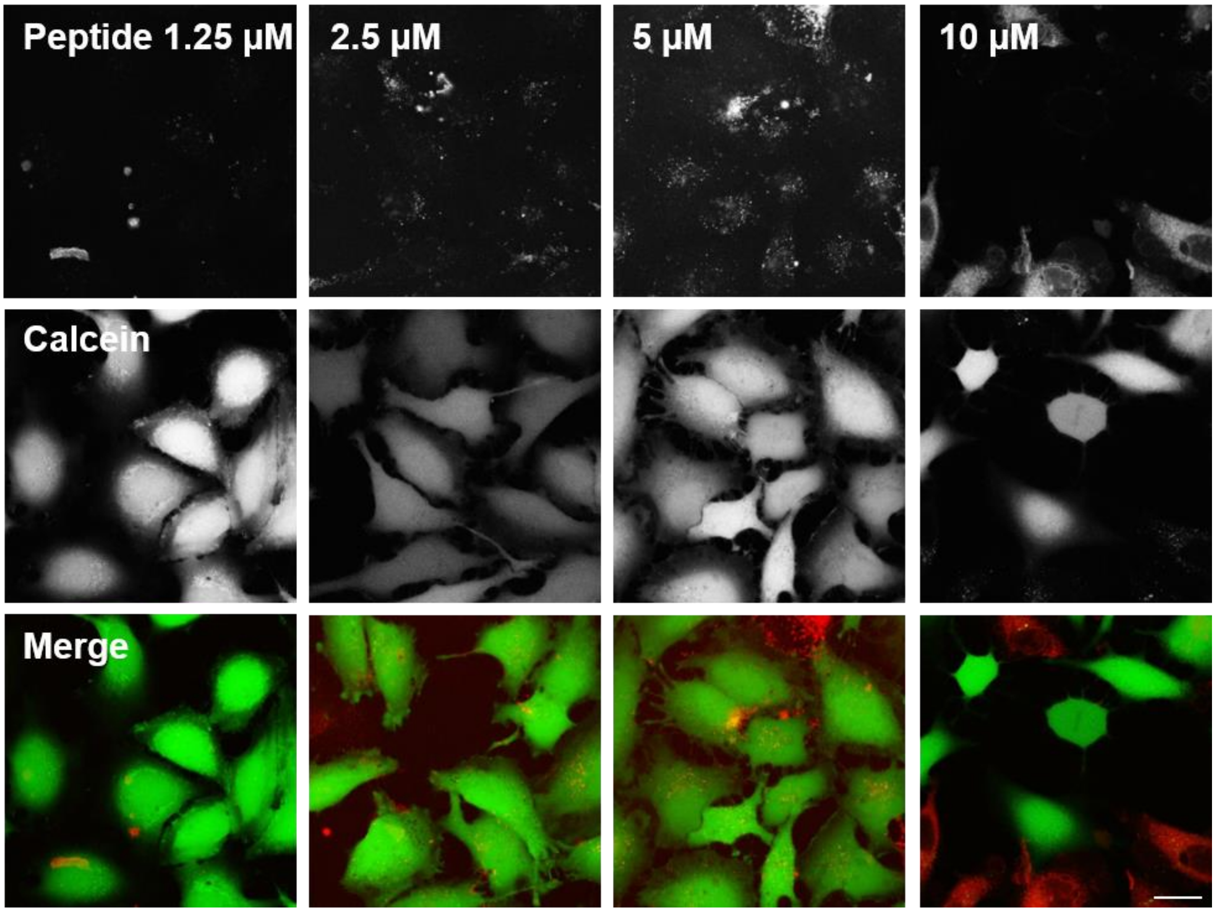

FIGURE S3. Cell uptake of PGLa in HeLa cells. Scale bar 25 µm.

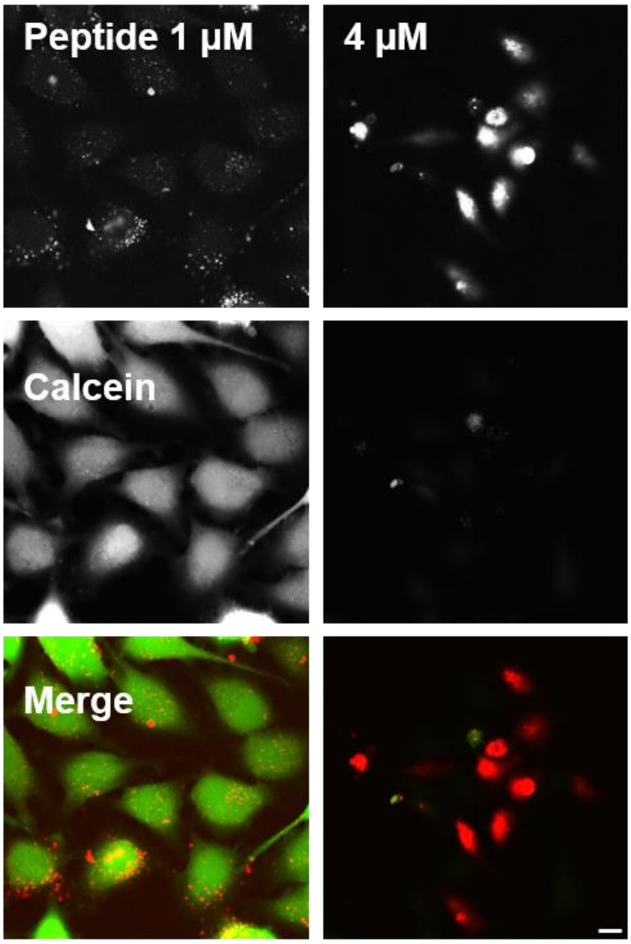

FIGURE S4. Cell uptake of MSI-103 in HeLa cells. Scale bar 25 µm.

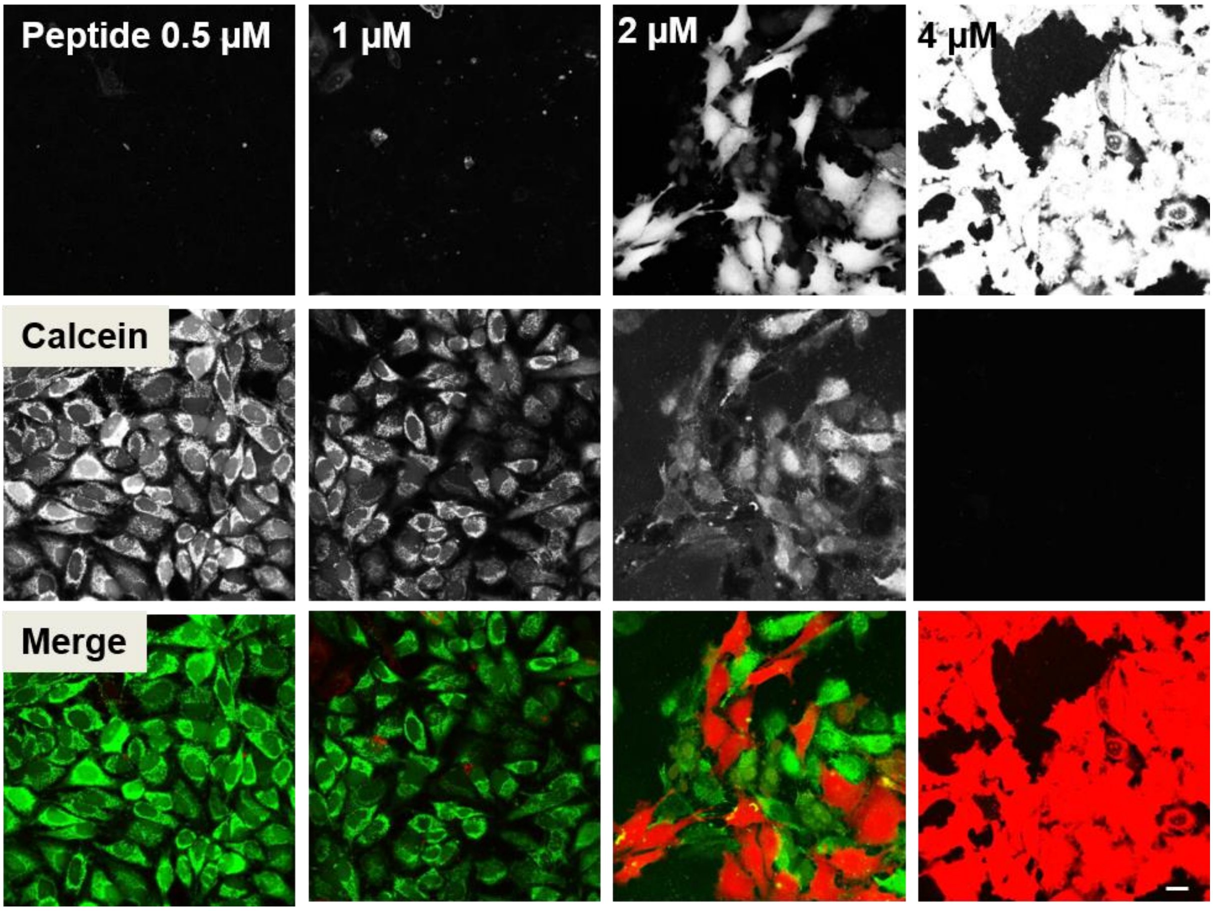

FIGURE S5. Cell uptake of MAP in HeLa cells. Scale bar 2.5 μm.

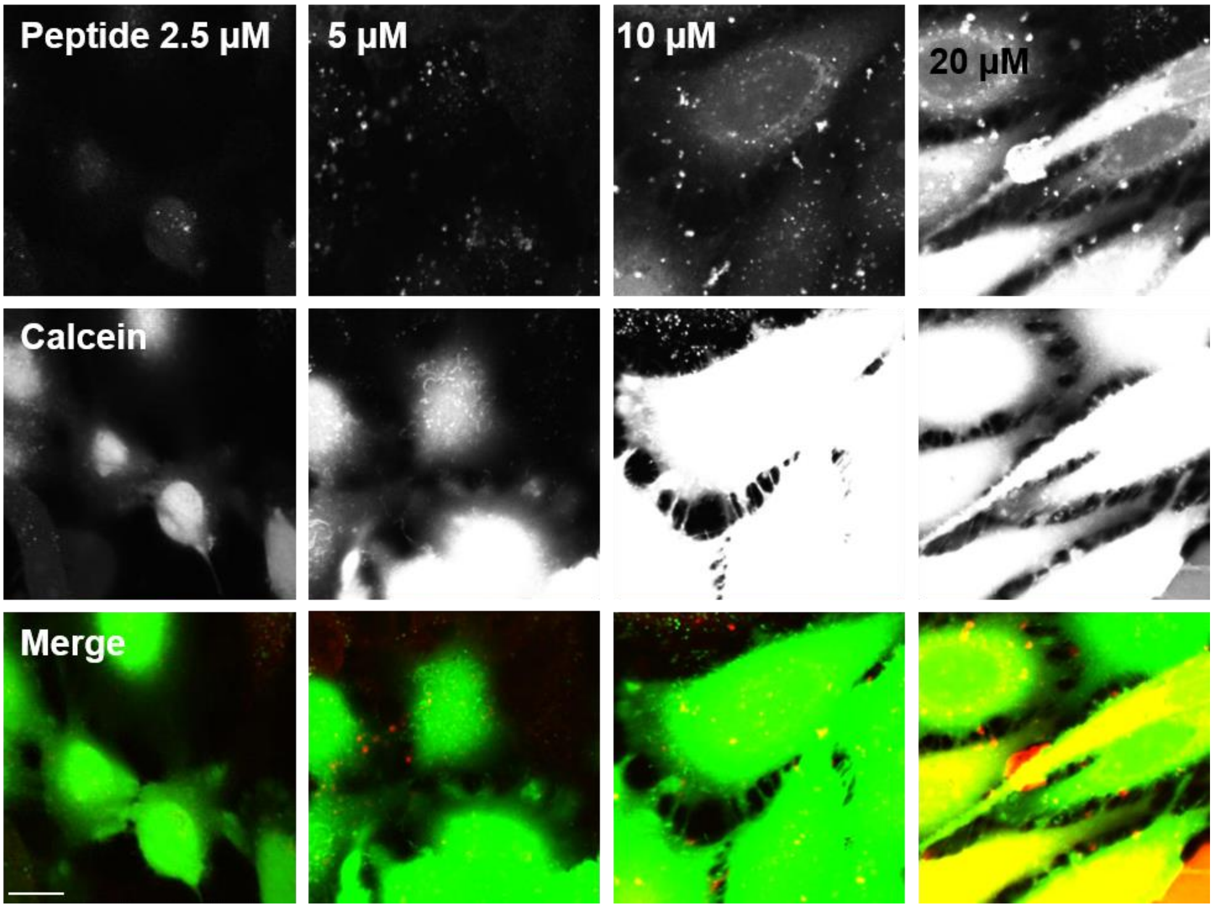

FIGURE S6. Cell uptake of TAT in HeLa cells. Scale bar 10 µm.
